# Pepper Bacterial Spot Control by *Bacillus velezensis*: Bioprocess Solution

**DOI:** 10.3390/microorganisms8101463

**Published:** 2020-09-24

**Authors:** Ivana Pajčin, Vanja Vlajkov, Marcus Frohme, Sergii Grebinyk, Mila Grahovac, Marija Mojićević, Jovana Grahovac

**Affiliations:** 1 Faculty of Technology, University of Novi Sad, Bulevar cara Lazara 1, 21000 Novi Sad, Serbia; vanja.vlajkov@uns.ac.rs; 2 Technical University of Applied Sciences Wildau, Hochschulring 1, 15745 Wildau, Germany; marcus.frohme@th-wildau.de (M.F.); sergii.grebinyk@th-wildau.de (S.G.); 3 Faculty of Agriculture, University of Novi Sad, Trg Dositeja Obradovića 8, 21000 Novi Sad, Serbia; mila@polj.uns.ac.rs; 4 Institute of Molecular Genetics and Genetic Engineering (IMGGE), University of Belgrade, Vojvode Stepe 444a, P. Fah 23, 11010 Belgrade, Serbia; marijamojicevic@imgge.bg.ac.rs

**Keywords:** *Xanthomonas euvesicatoria*, biological control, biocontrol agent, medium optimization, lipopeptides, fengycin, locillomycin, HPLC-MS, bioreactor, laboratory scale

## Abstract

Pepper bacterial spot is one of the most severe plant diseases in terms of infection persistence and economic losses when it comes to fresh pepper fruits used in nutrition and industrial processing. In this study, *Bacillus velezensis* IP22 isolated from fresh cheese was used as a biocontrol agent of pepper bacterial spot, whose main causal agent is the cosmopolitan pathogen *Xanthomonas euvesicatoria*. After optimization of the cultivation medium composition aimed at maximizing of the antimicrobial activity against *X. euvesicatoria* and validation of the optimized medium at the scale of a laboratory bioreactor, in planta tests were performed. The results have showed significant suppression of bacterial spot symptoms in pepper plants by the produced biocontrol agent, as well as reduction of disease spreading on the healthy (uninoculated) pepper leaves. Furthermore, HPLC-MS (high pressure liquid chromatography–mass spectrometry) analysis was employed to examine antimicrobial metabolites produced by *B. velezensis* IP22, where lipopeptides were found with similar *m*/*z* values compared to lipopeptides from fengycin and locillomycin families. The bioprocess solution developed at the laboratory scale investigated in this study represents a promising strategy for production of pepper bacterial spot biocontrol agent based on *B. velezensis* IP22, a food isolate with a great perspective for application in plant protection.

## 1. Introduction

*Xanthomonas euvesicatoria* is a bacterial phytopathogen and a cosmopolitan causal agent of pepper bacterial spot in countries with continental, tropical, and subtropical climate. Crop losses are usually very severe if the infection occurs at an early growth stage [1]. Defoliation as an infection consequence results in excessive sun exposure and possible burns to fruits [2]. Although these lesions are usually superficial, they still contribute to the lower market value of the fruits. The pathogen spreads mostly through the pepper seed and infected crop remains, but also by the rain and overhead irrigation [1]. *X. euvesicatoria* can survive in the soil, mostly in the rhizosphere of the non-host plant, as well as on pepper seeds for as long as 10 years [3]. Heavy rains, high air humidity, and temperature in the range 30–35 °C contribute to bacterial spot spreading [4,5]. Leaf infection occurs due to phytopathogen penetration in the plant tissue through the stomata and hydatodes, while the fruit infection happens in the wounded spots, such as abrasions and insect lesions. Phytopathogen can multiply epiphytically on young plants without visible disease symptoms [6]. Bacterial spot symptoms on pepper fruits are rarely observed because young infected fruits usually fall of. If there are any symptoms, they include scab-like, raised, and whitish lesions on fruits [2]. Leaf symptoms are more severe and include water-soaked irregular-shaped spots which become brownish and necrotic, and usually are surrounded by the large chlorotic zones, causing premature defoliation [7]. Bacterial spot of pepper has been reported in many European countries, as well as in USA, South America, Australia, and several Asian countries [8]. Disease severity implies an infection ratio as high as 50–95% [9], with significant economic losses due to necessity to remove infected plants and fruits in order to prevent repeated occurrence of the infection in the same field, which also represents a significant loss for the pepper processing industry and end-consumers of fresh pepper fruits.

Good agricultural practice when it comes to pepper bacterial spot consists of the use of healthy seed and transplants, as well as elimination of infected crop remains—application of phytosanitary measures [10]. Disease suppression methods include chemical treatments using copper-based bactericides in combination with ethylene bis-dithiocarbamates, but frequent application of these preparations has led to emergence of resistant *X. euvesicatoria* strains [11]. Moreover, these measures are not as effective as desired, even in the case of highly susceptible strains. In some countries where application of antibiotics in agriculture is allowed, streptomycin and kasugamycin have been used for suppression of *X. euvesicatoria*, with increased risk of development of the pathogen’s resistance [12]. Alternative strategies for bacterial spot management include application of plant resistance activators, such as acibenzolar-S-methyl [13], as well as application of biological control agents such as bacteriophages [14] and bacterial biocontrol agents [15].

When it comes to use of microorganisms or their metabolites in biological control of plant pathogens, bacteria of the genus *Bacillus* are the most commonly used microorganisms. Beneficial traits making them suitable for application in biological control include adaptability to different ecological conditions, short generation time, sporulation ability [16], and the ability to produce wide spectra of metabolites, such as antibiotics [17], enzymes [18], and biosurfactants [19]. Furthermore, *Bacillus* strains are usually well adapted to the conditions where they should be applied in the form of biopesticide, since their main habitat is soil [20]. *Bacillus velezensis* is one of the relatively novel species consisting of many strains with pronounced biocontrol traits after recent reclassification of strains from other closely related species based on comparative genomics [21]. Many *B. velezensis* strains present promising biocontrol agents due to ability to synthesize different antimicrobial metabolites, such as lipopeptides [22,23], enzymes [24], biosurfactants [25,26], and volatile organic compounds [27]. Moreover, these strains express remarkable plant and rhizosphere colonization ability, which is closely related to their plant growth-promotion capabilities [28] and induction of systemic resistance in plants [29].

However, most of the research studies are focused on isolation and screening of strains potent for biological control of different plant pathogens, as well as on determination of their mechanisms of antimicrobial and biocontrol action. Very few studies are oriented to development of biotechnological processes for production of biocontrol agents. In this study, *Bacillus* IP22 isolated from fresh cheese was investigated as a biocontrol agent of pepper bacterial spot caused by *X. euvesicatoria* strains. After optimization of the cultivation medium composition for production of biocontrol agents and validation experiment in the laboratory-scale bioreactor, the obtained cultivation broth was used for treatment of pepper plants in order to assess biocontrol activity against bacterial spot causers in planta. Furthermore, identification of *Bacillus* IP22 was performed using 16S rDNA sequencing, while analysis of the produced antimicrobial lipopeptides in the cultivation broth was carried out by HPLC-MS (high pressure liquid chromatography–mass spectrometry).

## 2. Materials and Methods

### 2.1. Microorganisms

*Bacillus* IP22 was isolated from fresh cheese and identified by 16S rDNA sequencing and kept on nutrient agar (HiMedia Laboratories, Mumbai, India) slant. Phytopathogenic *Xanthomonas* strains were isolated from diseased pepper plants with symptoms of bacterial spot. The pepper leaves were collected at several locations in the cadastral municipality Pivnice, Serbia. *Xanthomonas* isolates were kept on YMA (yeast maltose agar) slants containing 15 g/L of glucose, 5 g/L of peptone, 3 g/L of malt extract, 3 g/L of yeast extract and 20 g/L of agar. The isolates were identified by the PCR (polymerase chain reaction) method using species-specific primers (XeF and XeR) [30]. All microorganisms were subcultured on previously defined media and incubated during 48 h at 28 °C for *Bacillus* sp. and 26 °C for *Xanthomonas* spp. in order to regain physiological activity prior to further utilization.

### 2.2. 16S rDNA Sequencing and Identification of Bacillus IP22

*Bacillus* IP22 was cultured in 20 mL of tryptone soya broth medium (Difco Laboratories, Detroit, MI, USA) for 96 h with shaking at 30 °C. Cells were harvested by centrifugation (15 min, 5000 rpm) and biomass precipitate was resuspended in the solution for cell lysis (10 mL) containing sucrose (0.3 M), EDTA (25 mM), Tris-HCl (25 mM), RNase (2 U), whose pH value was set to 7.5. Incubation of bacterial suspension with lysozyme (10 mg) was performed at 37 °C for 30 min. After addition of proteinase K (5 mg, Sigma Aldrich, St. Louis, MO, USA) and 1 mL of 10% (*w*/*v*) SDS, incubation was carried out at 55 °C for 90 min. Afterwards, 15 mL of chloroform and 3.6 mL of 5 M NaCl were added with end-over-end rotation (6 rpm) for 20 min. Cell debris were removed by centrifugation (20 min, 5000 rpm). Precipitation of DNA from the supernatant was performed using isopropanol (1:1, *v*/*v*). DNA precipitate was rinsed with 70% (*v*/*v*) ethanol (1 mL), air dried, and dissolved in a buffer (10 mM Tris-HCl, 10 mM EDTA, pH 7.4), pre-warmed at 60 °C [31].

The 16S rDNA sequence was amplified from genomic DNA using universal bacterial primers 27F (5′-AGAGTTTGATCCTGGCTCAG-3′) and 1492R (5′-GGTTACCTTGTTACGACTT-3′) [32]. PCR amplification was performed in 2720 Thermal Cycler (Applied Biosystems, Thermo Fisher Scientific, Waltham, MA, USA) using FASTA Gene TAQ PCR kit following the manufacturer’s protocol. PCR products were purified using a PCR purification kit (Qiagen, Hilden, Germany). Sequencing was performed with a BigDye™ Terminator v3.1 Cycle Sequencing kit (Applied Biosystems, Thermo Fisher Scientific, Waltham, MA, USA) on the Applied Biosystems 3130 Genetic Analyzer (Applied Biosystems, Thermo Fisher Scientific, Waltham, MA, USA). 16S rDNA sequences were identified using BLASTN program. Evolutionary analyses were conducted in MEGA7 [33]. 16S rDNA sequence of *Bacillus* IP22 has been deposited in the GenBank repository under accession number MT883432.

### 2.3. PCR Identification of Xanthomonas spp.

*Xanthomonas* spp. were identified using PCR according to the procedure given by Moretti et al. [30] using the species-specific primers for *X. euvesicatoria* – XeF (5′-CTGGGAAACTCATTCGCAGT-3′) and XeR (5′- TTGTGGCGCTCTTATTTCCT-3′). Two referent isolates were used as positive controls (*X. euvesicatoria* 5 and *X. euvesicatoria* ref 1). These referent isolates were obtained from the culture collection of the Laboratory for seed testing of the Institute of Field and Vegetable Crops (Novi Sad, Serbia). DNA extraction from the tested and referent isolates, PCR reaction, and visualization of the obtained PCR products took place at the same laboratory. Pathogenic isolates were grown on nutrient agar (HiMedia, Mumbai, India; 26 °C, 24 h) and a single colony for each isolate was picked and suspended in sterile distilled water (100 µL) in microtubes. Sterile distilled water was used as negative control. Cell lysis was performed according to the following procedure: heating at 95 °C for 15 min, cooling on ice, and centrifugation in order to separate DNA (11,000 rpm, 5 min).

PCR mixture (25 µL) consisted of 12.5 µL of 2× MMix (Eppendorf, Hamburg, Germany), 0.5 µL of 10 μM Forward Primer, 0.5 µL of 10 μM Reverse Primer, 2.0 µL of template DNA, and 9.5 µL of nuclease-free water. PCR reaction conditions are described in Table S1.

The obtained PCR products (10 µL) were separated using gel-electrophoresis on agarose gel (1.5% *w*/*v*) in 1xTBE (Tris-borate-EDTA) buffer (30 min, constant voltage of 100 V, maximal current of 5 V/cm). Appearance of 208 bp-fragments during observation by UV transluminator was considered as a positive reaction for identification of the pathogens as members of the *X. euvesicatoria* species.

### 2.4. Modeling and Optimization of Cultivation Medium Composition

Optimization of medium composition for cultivation of the producing microorganism *Bacillus* IP22 was performed to determine optimal content of the following nutrients: glycerol as carbon source, yeast extract as organic nitrogen source, (NH_4_)_2_SO_4_ as inorganic nitrogen source, and K_2_HPO_4_ as phosphorus source. Cultivation media were prepared according to the Box–Behnken 3^4^ experimental plan (Table S2), where four independent variables were varied at three levels: glycerol concentration 10–35–60 g/L, yeast extract concentration 0–2.5–5 g/L, (NH_4_)_2_SO_4_ concentration 0–1.5–3 g/L, and K_2_HPO_4_ concentration 1–5.5–10 g/L. Besides previously mentioned nutrients, each cultivation medium has contained MgSO_4_∙7H_2_O (0.3 g/L). All media were sterilized by autoclaving (121 °C, 2.1 bar, 20 min), while pH value of each medium was adjusted to 7.0 ± 0.2 prior to sterilization.

Inoculum was prepared using nutrient broth (HiMedia, Mumbai, India). *Bacillus* IP22 was cultivated on a laboratory shaker (150 rpm, 28 °C, 48 h, spontaneous aeration). Inoculum volume was 10% (*v*/*v*) compared to cultivation medium volume (50 mL). After inoculation, cultivation was carried out in Erlenmayer flasks on a laboratory shaker (150 rpm, 28 °C, 96 h, spontaneous aeration).

Dependent variables for modeling and optimization of cultivation medium composition were antimicrobial activity of cultivation broth samples (measured as inhibition zone diameter against *Xanthomonas* spp.) and residual content of the main nutrients (carbon, nitrogen, and phosphorus). After the end of cultivation, cultivation broth samples were used for antimicrobial activity testing using the diffusion-disc method. Residual content of nutrients (glycerol, total nitrogen, and total phosphorus) was determined using supernatants obtained after centrifugation of cultivation broth samples (10,000 rpm, 10 min; Rotina 380R, Hettich, Kirchlengern, Germany). Four second-degree polynomial equations used as models for optimization of cultivation medium composition were obtained using the Statistica 13.3 software (Dell Technologies, Round Rock, TX, USA). The same software was used to generate response surfaces graphs in order to better understand interactions between the input variables and their effect to inhibition zone diameter as the main indicator of antimicrobial activity. Optimization of the cultivation medium composition was performed using the desirability function method in the DesignExpert 8.1. software (Stat-Ease, Inc., Minneapolis, MN, USA).

### 2.5. Validation Experiment

Validation experiment was performed in the laboratory-scale bioreactor (Biostat^®^ Aplus, Sartorius AG, Göttingen, Germany) using the cultivation medium of an optimized composition. Working volume of the bioreactor was 2 L, while inoculum volume was 10% (*v*/*v*) compared to the cultivation medium volume. Inoculum was prepared in the same way as in the previous stage of the experiments. Cultivation of *Bacillus* IP22 was carried out at 28 °C, with agitation using Rushtone turbine with three impellers (agitation rate 250 rpm), under aerobic conditions using sterile air for aeration (aeration rate 1 vvm – volume of air/(volume of liquid∙min)). During 96 h of cultivation, the following parameters were continuously measured: pH value and temperature. At the defined time intervals (12 h), cultivation broth was sampled from the bioreactor to determine biomass content, antimicrobial activity of the cultivation broth samples, as well as residual content of glycerol, total nitrogen, and total phosphorus. Biomass content was measured using DCW (dry cell weight) and spectrophotometric measurement of optical density of cultivation broth samples. Validation experiment was carried out in triplicate tests.

### 2.6. In Planta Experiments

The resulting *Bacillus* IP22 cultivation broth obtained after the validation experiment was used to assess potential of the produced biocontrol agents in biological control of bacterial spot of pepper. Pepper plants (sort Blancina) were obtained from a commercial nursery garden (Grow rasad d.o.o., Irig, Serbia). The following variants were assessed: uninoculated and untreated control, positive control (untreated plants inoculated with *Xanthomonas* strains PL1 and PL2), plants treated with *Bacillus* IP22 cultivation broth and inoculated with *Xanthomonas* strains PL1 and PL2 (Table 1).

Plants were grown in pots with a diameter of 9 cm and height of 8 cm, using the substrate Pindstrup Plus Blue (pH value 6.0) (Pindstrup Moseburg A/S, Ryomgaard, Denmark). Treatment with *Bacillus* IP22 cultivation broth was performed by dipping the plants in the cultivation broth. On the other hand, suspensions of *Xanthomonas* isolates were prepared using sterile saline to achieve 10^8^ CFU/mL. Inoculation of plants was performed 24 h after the treatment using the sterile syringe (except for positive controls, where treatment with *Bacillus* IP22 cultivation broth was not performed). Two leaves were inoculated in each plant, with three injuries made at each leaf in the area between the leaf veins. Plants were kept in closed separate transparent containers (length 0.6 m, width 0.4 m, height 0.4 m) at 25 °C, exposed to sunlight in regular daily intervals (on average 14 h/day), in humid atmosphere (relative air humidity 92.0 ± 5.0) for 4 weeks. No additional supplementation of plants with nutrients was performed during the in planta experiment. Afterwards, the diameters of the emerged lesions were measured and the results were expressed as necrosis area relative to leaf area. In addition, the number of leaves with symptoms of bacterial spot per plant was recorded. Each variant contained 8 plants, and in planta assay was performed in triplicate tests.

### 2.7. Analytical Methods

#### 2.7.1. In Vitro Antimicrobial Activity Assaying

Antimicrobial activity of cultivation broth samples obtained after the cultivation of *Bacillus* IP22 against the phytopathogenic *Xanthomonas* isolates PL1 and PL2 was assessed using the diffusion-disc method. Suspensions of *Xanthomonas* isolates were prepared using sterile saline and used for inoculation of melted and tempered (50 ± 1 °C) YMA medium, which was poured in Petri dishes (90 mm) after the inoculation. After solidification of the medium, three paper discs (HiMedia, Mumbai, India) were placed in each Petri dish. Volume of the tested *Bacillus* IP22 cultivation broth samples applied to the discs was 15 μL. Negative control was sterile distilled water. Incubation was carried out at 26 °C for 72 h, upon which inhibition zone diameters were measured.

#### 2.7.2. Determination of Residual Content of Nutrients

Residual content of the main nutrients (glycerol, total nitrogen, and total phosphorus) was determined using the biomass-free supernatants obtained by centrifugation of samples of *Bacillus* IP22 cultivation broth. Glycerol content was determined using the HPLC method. The HPLC system (Thermo Scientific Dionex UltiMate 3000 series; Thermo Fisher Scientific, Waltham, MA, USA) consists of the following components: pump HPG-3200SD/RS, autosampler WPS-3000(T)SL (10 μL injection loop), column Zorbax NH2 (250 mm × 4.6 mm, 5 μm; Agilent Technologies, Santa Clara, CA, USA), and the refractive index detector (ERC RefractoMax520, ERC GmbH, Riemerling, Germany). Mobile phase was 70% (*v*/*v*) acetonitrile. Analysis parameters were: mobile phase flow rate 1 mL/min, run time 15 min, column temperature 30 °C, detector temperature 45 °C, injection volume 10 μL.

Residual content of total nitrogen was determined by the Kjeldahl method [34], while residual content of total phosphorus was determined using the spectrophotometric method with ascorbic acid [35].

#### 2.7.3. Determination of *Bacillus* IP22 Biomass Content

Dry cell weight of *Bacillus* IP22 biomass was measured using the pellets obtained after centrifugation of the cultivation broth (20 mL, 10,000 rpm, 10 min) and decanting of the supernatant. The obtained biomass pellets were dried at 105 °C until reaching the constant weight. Biomass concentration in cultivation broth was expressed in g/L as DCW (dry cell weight) in 20 mL of cultivation broth.

Measurement of optical density of *Bacillus* IP22 cultivation broth was performed at wavelength of 600 nm using the spectrophotometer (UV-1800, Shimadzu, Kyoto, Japan). The blank was cultivation medium used for cultivation of *Bacillus* IP22 in the laboratory-scale bioreactor.

#### 2.7.4. HPLC-MS Analysis of the Antimicrobial Lipopeptides Produced by *Bacillus* IP22

Sample used for analysis of the antimicrobial lipopeptides produced by *Bacillus* IP22 was the supernatant obtained after centrifugation of cultivation broth sample at the end of the validation experiment. HPLC-MS was used for lipopeptide analysis as proposed by Smyth et al. [36].

Mass spectrometry analysis after chromatographic separation was achieved with a tandem quadruple mass spectrometer LCMS-8040, equipped with an electrospray ionization (ESI) source (Shimadzu, Kyoto, Japan) coupled to a Nexera high-performance liquid chromatography (HPLC) system. HPLC separation was performed with XDB-C18 (100 mm × 4.6 mm, 3 μM) column (Agilent Technologies, Santa Clara, CA, USA). The column was thermostabilized at 40 °C. A mobile phase of 5 mmol/L ammonium acetate solution containing 0.1% (*v*/*v*) formic acid (A) and methanol (B) was used. The following linear gradient elution was used: 10% B held to 1 min, then increased to 90% from 1 to 7 min, then held at 90% B from 7 to 12 min, then decreased to 10% B from 12 to 13.5 min, and further held at 10% B until 16 min. The flow rate was set at 0.5 mL/min. The mass spectrometer was mass-calibrated against an autotuning standard solution (a mixture of PEG, PPG, and raffinose: *m*/*z* 65.05, 168.10, 256.15, 344.20, 652.40, 1004.60, and 1224.75) for LC–MS (Shimadzu, Kyoto, Japan). For mass detection, both positive and negative ionization modes were used. The technical parameters for the MS measurements were a spray capillary voltage of 3.0 kV, a detector voltage of 2.04 kV, an interface voltage of 4.5 kV, a desolvation line temperature of 250 °C, a heat block temperature of 450 °C, a nebulizing gas flow rate of 3.0 mL/min, a drying gas flow rate of 15 mL/min. Acquisition was performed in the selected ion monitoring (SIM) mode with a dwell time of 0.2 s.

### 2.8. Statistical Analysis of the Experimental Data

The experimental data obtained after the cultivation of *Bacillus* IP22 in the phase of modeling of cultivation medium composition were fitted using four polynomial equations of the second order for the following bioprocess responses: inhibition zone diameter, residual glycerol content, residual total nitrogen content, and residual total phosphorus content. Statistical analysis of the experimental data for modeling of cultivation medium composition was performed using the Statistica 13.3 software (Dell Technologies, Round Rock, TX, USA). All statistical analyses were performed at significance level of 95%. Mean values and standard deviations for the experimental replications during validation experiments were calculated and plotted to monitor cultivation course using Origin 9 software (OriginLab Corporation, Northampton, MA, USA). Mean values and standard deviations for replications in the phase of in planta testing were calculated using Microsoft Excel 2010 (Microsoft Corporation, Redmond, WA, USA). Duncan’s multiple range test was also performed using the results of in planta testing to establish homogenous groups of variances using the Statistica 13.3 software (Dell Technologies, Round Rock, TX, USA).

## 3. Results

### 3.1. 16S rDNA Sequencing and Identification of Bacillus IP22

16S rDNA sequence alignment and phylogenetic analysis (16S ribosomal DNA sequences Database) of the selected producing microorganism have revealed closest similarity to B*acillus velezensis* (100% query coverage, 99% homology).

**Figure 1:**
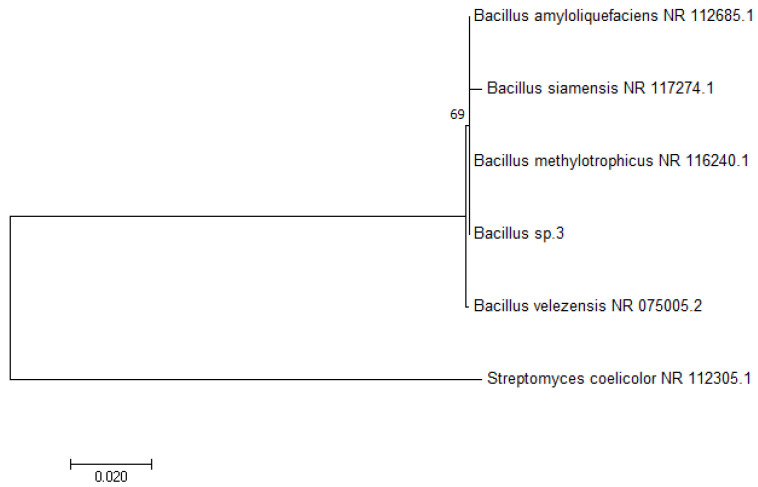
Molecular phylogenetic analysis of the *Bacillus* IP22 (in the figure marked as *Bacillus* sp.3) 16S rDNA sequence by the maximum likelihood method.

The Tamura–Nei model [37] and the maximum likelihood method were used to infer the evolutionary history. Figure 1 shows the phylogenetic tree with the highest log likelihood (−1378.78), while the numbers next to the branches represent the percentage of trees in which the associated taxa clustered together. Neighbor-join and BioNJ algorithms were applied to obtain initial tree(s) for the heuristic search, while the maximum composite likelihood (MCL) approach was applied to estimate the matrix of pairwise distances, followed by the selection of topology with the highest log likelihood value. The phylogenetic tree is drawn to scale, where branch lengths represent the number of substitutions per site, while six nucleotide sequences were included in the analysis. Included codon positions were 1st+2nd+3rd+Noncoding. Final dataset consisted of 661 positions, while elimination was performed on all positions which contained missing data and gaps.

Based on the presented results, the producing microorganism was identified as a member of the species *Bacillus velezensis*.

### 3.2. PCR Identification of Xanthomonas spp.

**Figure 2:**
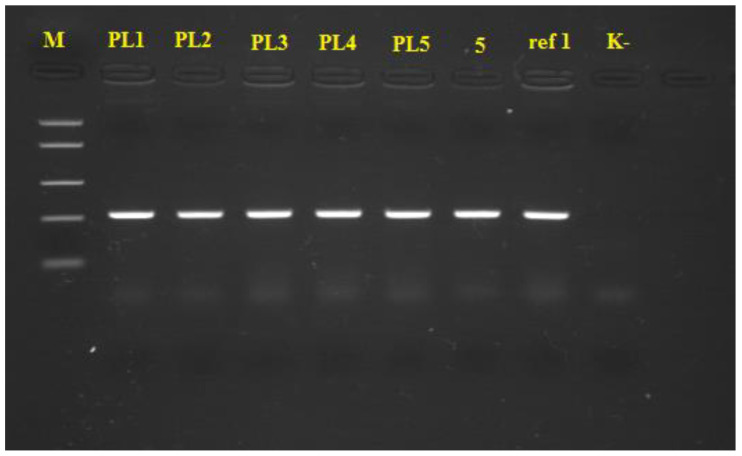
Visualization of the amplified PCR products in 1.5% agarose gel during PCR identification of pathogenic *Xanthomonas* spp. (M—gene ruler, PL1-PL5—PCR products from phytopathogenic *Xanthomonas* spp., 5 and ref 1—PCR products from referent isolates, K—negative control)

The results of visualization of the obtained PCR products are represented in Figure 2. PCR products with length of 208 bp obtained using species-specific primers were successfully amplified for all tested pathogenic isolates, as well as for referent *X. euvesicatoria* strains (5 and ref 1). There was no amplification of the selected DNA fragment in negative control. Based on the presented results, all pathogenic isolates from pepper leaves were identified as *X. euvesicatoria* strains. Isolates PL1 and PL2 were selected for further biocontrol trials.

### 3.3. Modeling and Optimization of Medium Composition for Cultivation of B. velezensis IP22

Cultivation medium used for cultivation of *B. velezensis* IP22, i.e., for production of biocontrol agent, has contained several nutrients (glycerol as carbon source, yeast extract as organic nitrogen source, (NH_4_)_2_SO_4_ as inorganic nitrogen source, and K_2_HPO_4_ as phosphorus source), whose effects on antimicrobial activity of the producing strain against phytopathogenic *X. euvesicatoria* strains have been investigated. Furthermore, besides the effect on inhibition zone diameter as the main indicator of antimicrobial activity of *B. velezensis* IP22 cultivation broth against *X. euvesicatoria*, initial content of these nutrients in the medium for cultivation of *B. velezensis* IP22 was varied (Table 2) in order to investigate their effects to residual content of carbon, nitrogen, and phosphorus in the resulting cultivation broth after the cultivation of *B. velezensis* IP22.

**Figure 3:**
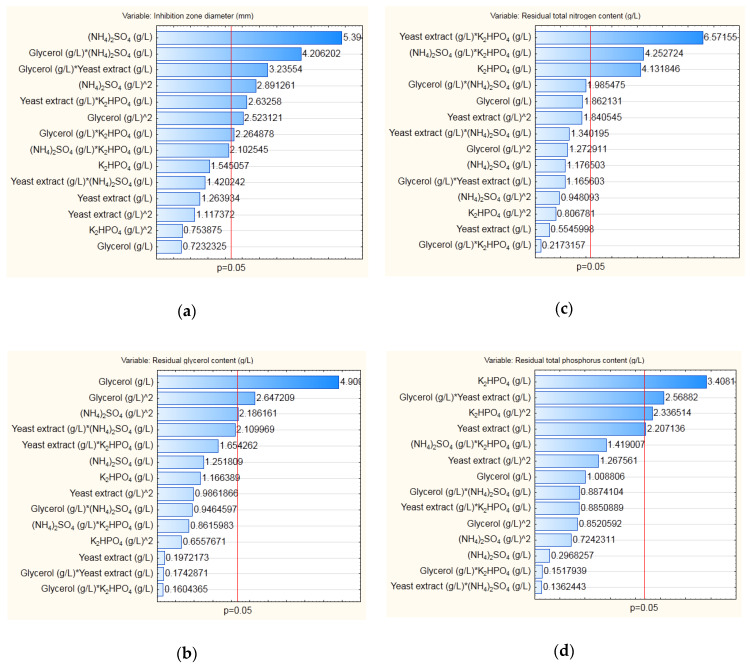
Pareto charts—coefficients of regression models for the selected dependent variables during modeling of medium composition for cultivation of *B. velezensis* IP22: (**a**) inhibition zone diameter, (**b**) residual glycerol content, (**c**) residual total nitrogen content, (**d**) residual total phosphorus content. The red line in the charts represents a limit of statistical significance (*p*-value equal to 0.05) at the confidence level of 95%.

For purpose of modeling, second degree polynomial equations were used to fit the experimental data. The obtained linear, quadratic, and interaction regression coefficients, as well as their statistical significance (*p*-value less than 0.05), are presented using Pareto charts for the selected dependent variables—inhibition zone diameter, residual content of glycerol, residual total content of nitrogen, and residual total content of phosphorus (Figure 3).

Analysis of variance (ANOVA) was also performed for each model obtained for the selected outputs in order to assess whether the obtained models are statistically significant with confidence level of 95% (Table 2).

Considering the calculated statistical parameters for analysis of variance, it can be concluded that each model was statistically significant with confidence level of 99%, with *p*-values lower than 0.01.

**Figure 4:**
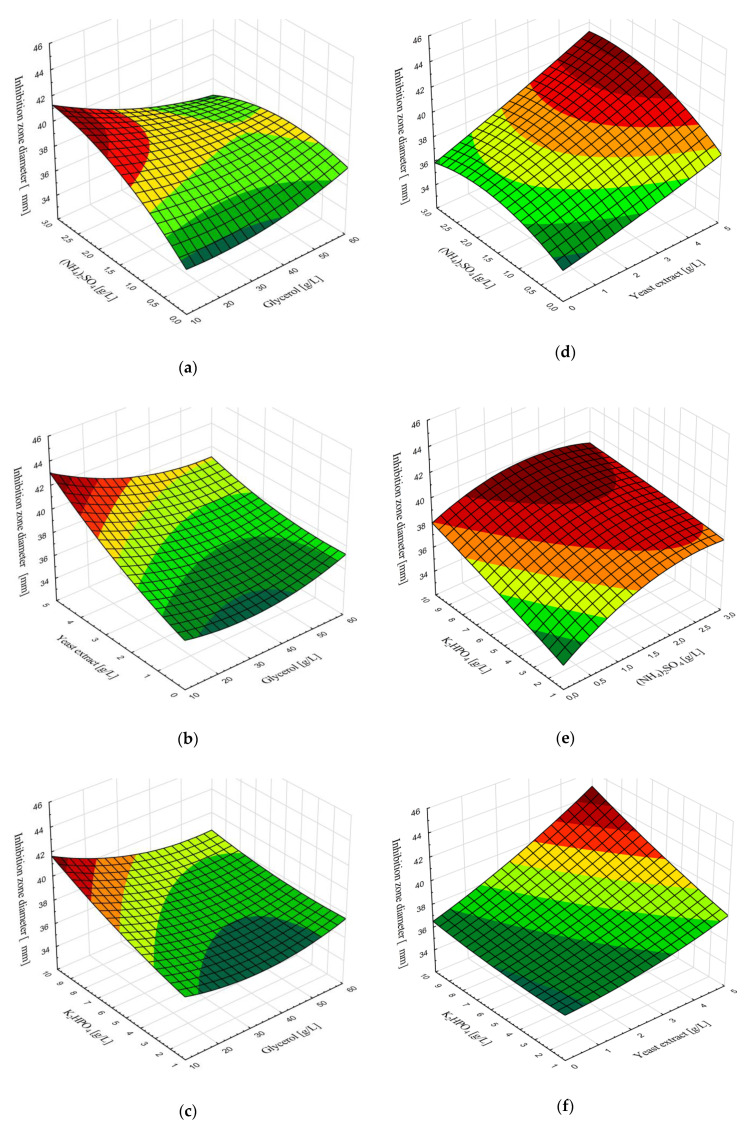
Response surface plots for effects of nutrients’ content on inhibition zone diameter for the following combination of independent variables: (**a**) glycerol and (NH_4_)_2_SO_4_, (**b**) glycerol and yeast extract, (**c**) glycerol and K_2_HPO_4_, (**d**) (NH_4_)_2_SO_4_ and yeast extract, (**e**) (NH_4_)_2_SO_4_ and K_2_HPO_4_, (**f**) yeast extract and K_2_HPO_4_.

In order to investigate the effect of nutrients’ content on inhibition zone diameter, as the dependent variable which mostly affects antimicrobial activity of *B. velezensis* IP22 against *X. euvesicatoria*, response surface plots (Figure 4) were generated to present the effects of two variables, while the other two remained constant at the value of central point from the Box–Behnken experimental plan (Table S2).

Considering the effects of main nutrients (glycerol, yeast extract, (NH_4_)_2_SO_4_, and K_2_HPO_4_), whose initial concentrations in the cultivation medium were selected as independent variables, on inhibition zone diameter, it could be concluded that maximal inhibition zone diameter against *X. euvesicatoria* is expected when using minimal concentration of glycerol (around 10 g/L), almost maximal concentration of yeast extract (4.5–5 g/L), ((NH_4_)_2_SO_4_ in the concentration range 1–3 g/L, and almost maximal concentration of K_2_HPO_4_ (9–10 g/L). The expected maximal inhibition zone diameter is in the range 42–45 mm (Figure 4).

Optimization of the medium composition for production of biocontrol agents by *B. velezensis* IP22, in terms of initial content of the main nutrients (glycerol as carbon source, yeast extract as organic nitrogen source, (NH_4_)_2_SO_4_ as inorganic nitrogen source, and K_2_HPO_4_ as phosphorus source), was the next step in bioprocess development. Optimization of cultivation medium composition was performed using the desirability function method, where the same importance coefficient was assigned to each independent and dependent variable, while desirability function was calculated using the DesignExpert software for the desired optimization outcomes defined as optimization aims (Table 3).

The first optimization set was aimed at maximization of antimicrobial activity of *B. velezensis* IP22 cultivation broth against *X. euvesicatoria*. Therefore, the goal of the first optimization set was to maximize inhibition zone diameter obtained by the antimicrobial activity testing. Optimization results have showed that maximal inhibition zone diameter of 66.75 mm could be obtained using the medium for cultivation of *B. velezensis* IP22 of the following composition: glycerol 12.0 g/L, yeast extract 4.0 g/L, (NH_4_)_2_SO_4_ 2.7 g/L, K_2_HPO_4_ 8.1 g/L, and MgSO_4_∙7H_2_O 0.3 g/L. Furthermore, desirability function value 1 has implied complete fulfillment of the optimization goal previously set. However, since usage of cultivation medium formulated as given in the first optimization set would result in very high residual content of carbon, nitrogen, and phosphorus in the cultivation broth at the end of cultivation, one more optimization set was performed to minimize residual content of the aforementioned nutrients, simultaneously with keeping the antimicrobial activity maximized. Desirability function value of 0.77 has implied satisfying fulfillment of the optimization goals using the cultivation medium of the following composition: glycerol 10.0 g/L, yeast extract 2.8 g/L, (NH_4_)_2_SO_4_ 3.0 (g/L), K_2_HPO_4_ 1.0 (g/L), and MgSO_4_∙7H_2_O 0.3 g/L.

### 3.4. Validation Experiment–Cultivation of B. velezensis IP22 in a Laboratory-Scale Bioreactor

**Figure 5:**
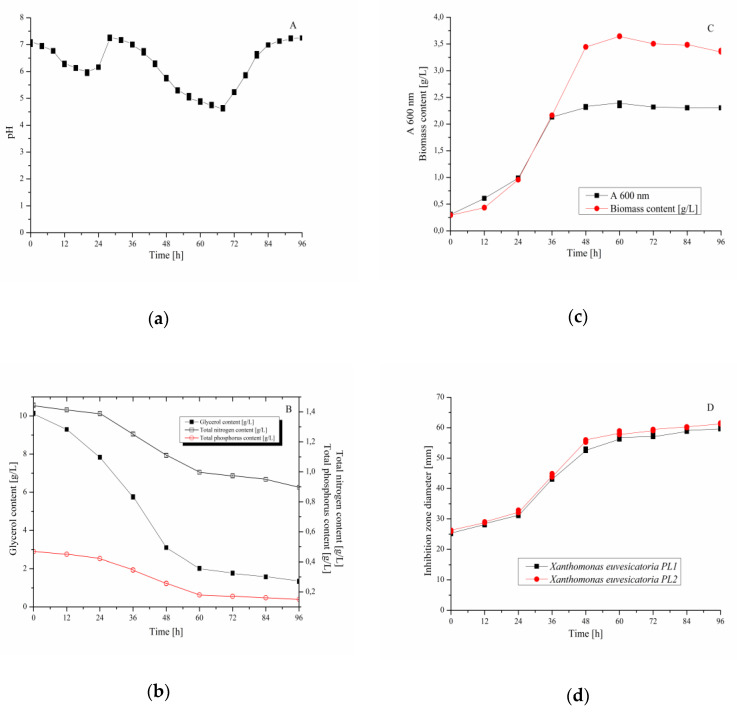
Course of *B. velezensis* IP22 cultivation in the laboratory-scale bioreactor in terms of: (**a**) pH value, (**b**) residual nutrients’ content, (**c**) biomass content, (**d**) antimicrobial activity against *X. euvesicatoria* isolates.

Experiment aimed at validation of the optimized medium composition for cultivation of *B. velezensis* IP22 was performed in the laboratory-scale bioreactor, as a bioprocess vessel of a larger volume (working volume of 2 L). During cultivation, temperature, mixing, and aeration regulation were performed to keep constant temperature (28 °C), agitation rate (250 rpm) and aeration rate (1 vvm). Bioprocess parameters that were monitored during the cultivation were pH value (Figure 5a) and temperature. Temperature was maintained constant (28 °C) during the whole bioprocess due to temperature regulation. Cultivation broth samples were sampled at predefined time intervals (12 h) in order to determine biomass content (concentration of *B. velezensis* IP22 biomass and optical density of the cultivation broth) and residual nutrients’ content (glycerol, total nitrogen, and total phosphorus), as well as antimicrobial activity of cultivation broth samples against *X. euvesicatoria*. Cultivation course considering these variables is given in Figure 5. The results represent mean values and standard deviation from three cultivations performed under the similar conditions in the laboratory-scale bioreactor.

**Figure 6:**
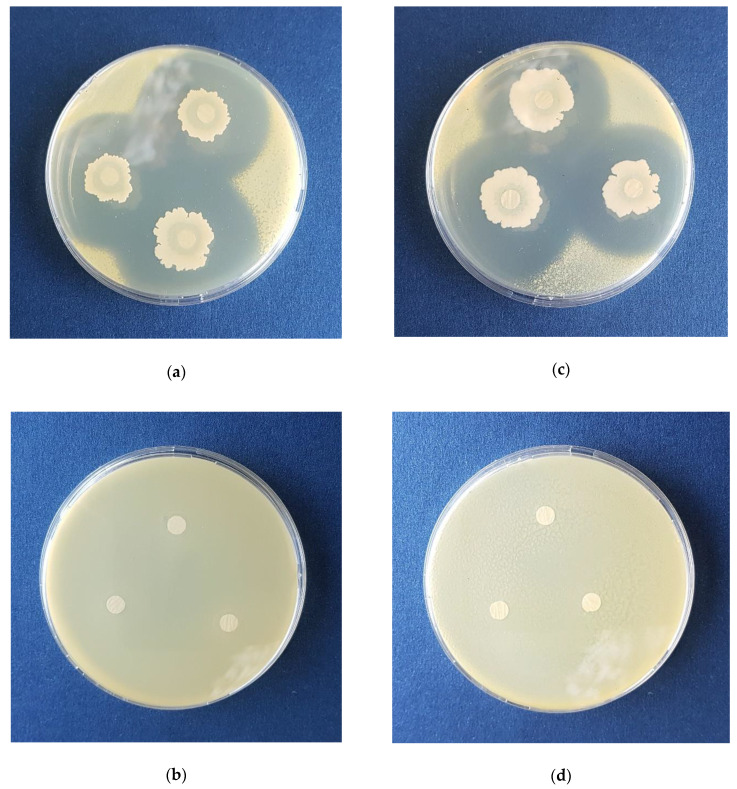
Inhibition zone diameters obtained as a result of in vitro antimicrobial activity testing against *X. euvesicatoria*: (**a**) cultivation broth sample from the end of the validation experiment against *X. euvesicatoria* PL1, (**b**) negative control—*X. euvesicatoria* PL1, (**c**) cultivation broth sample from the end of the validation experiment against *X. euvesicatoria* PL2, (**d**) negative control—*X. euvesicatoria* PL2.

When it comes to residual concentration of the main nutrients (glycerol, total nitrogen, and total phosphorus) during the cultivation, it can be noticed that in the first 24 h of cultivation, slight decrease of nutrients’ content had occurred (Figure 5b) due to adaptation of *B. velezensis* IP22 from metabolizing sugars from nutrient broth used for inoculum preparation to glycerol as the main carbon source of cultivation medium in the bioreactor [38]. Sharper decrease in the nutrients’ content could be observed between the 24th and the 60th hour (Figure 5b) due to the exponential growth phase, which could also be observed in Figure 5c, where biomass content has drastically increased in the same period of the cultivation. After 60 h of cultivation, only slight change in nutrients’ content, as well as in biomass content, was noticed, corresponding to the stationary growth phase, where the number of newly formed bacterial cells is approximate to the number of dying bacterial cells, which is also confirmed by the stagnation of optical density of the cultivation broth (Figure 5c). Monitoring of inhibition zone diameters (Figure 5d) against *X. euvesicatoria* has suggested that the trend of their change has approximately followed the trend of biomass content change, as well as the trend of nutrients’ consumption. The results of antimicrobial activity testing using the cultivation broth sample from the end of the validation experiment (96th hour) against X. *euvesicatoria* PL1 and PL2 are given in Figure 6.

### 3.5. HPLC-MS Analysis of Antimicrobial Compounds Produced by B. velezensis IP22

**Figure 7:**
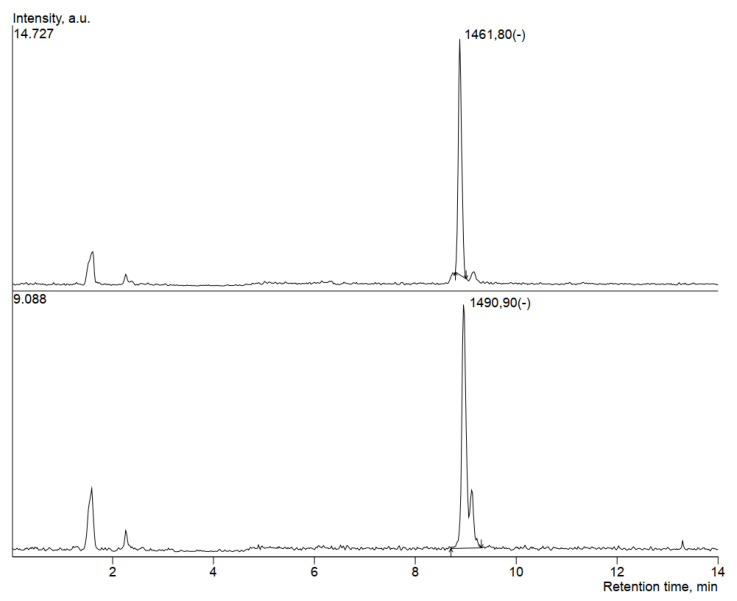
SIM (selected ion monitoring) chromatograms of the antimicrobial compounds from the fengycin family putatively identified in the supernatant of *B. velezensis* IP22 cultivation broth.

**Figure 8:**
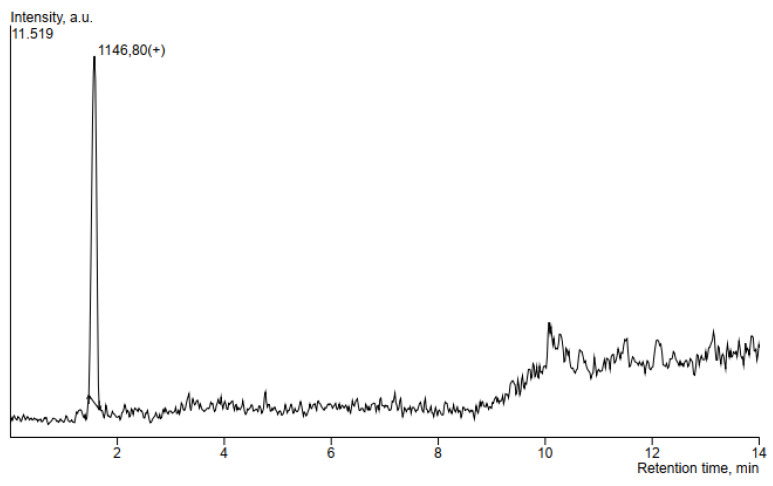
SIM chromatogram of the antimicrobial compound from the locillomycin family putatively identified in the supernatant of *B. velezensis* IP22 cultivation broth.

In order to determine the ability of the producing microorganism *B. velezensis* IP22 to produce antimicrobial lipopeptides, HPLC-MS analysis was performed using the supernatant obtained after centrifugation of the cultivation broth sample from the end of the validation experiment. The obtained SIM chromatograms are given in Figure 7 and Figure 8, while the obtained *m*/*z* values of the produced antimicrobial compounds were compared to literature data. The obtained results are summarized in Table 4.

In this study, compounds identified at *m*/*z* values of 1461.8, 1489.7, and 1490.9 suggest the presence of fengycins in the supernatant of cultivation broth obtained by cultivation of *B. velezensis* IP22 (Figure 7) [39,40,41,42,43,44].

The detected mass in 1146.8 (Figure 8) pointed to the presence of locillomycin, a relatively new lipopeptide family that has been synthesized by the bacteria of the genus *Bacillus* [45].

### 3.6. In Planta Experiments with Pepper Plants

**Figure 9:**
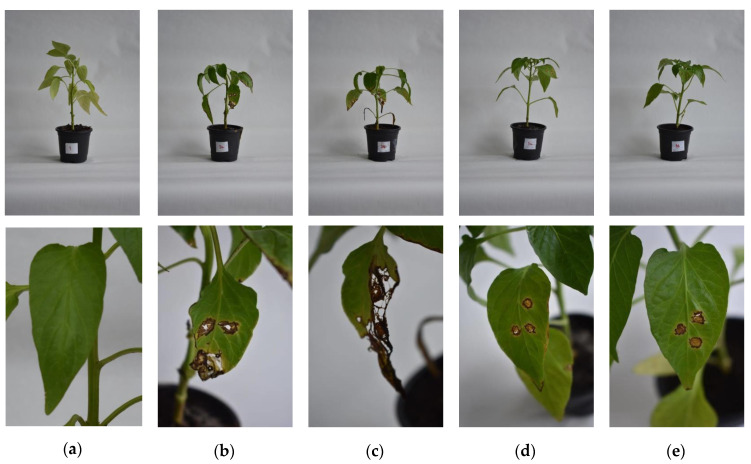
The results of in planta testing of the produced biocontrol agents based on *B. velezensis* IP22 against *X. euvesicatoria*: (**a**) uninoculated and untreated control (negative control—plant 1), (**b**) untreated control inoculated with *X. euvesicatoria* PL 1 (positive control—plant 2A), (**c**) untreated control inoculated with *X. euvesicatoria* PL 2 (positive control—plant 2B), (**d**) plant treated with *B. velezensis* cultivation broth and inoculated with *X. euvesicatoria* PL 1 (plant 3A), (**e**) plant treated with *B. velezensis* cultivation broth and inoculated with *X. euvesicatoria* PL 2 (plant 3B).

In planta testing of biocontrol activity in pepper plants was performed using the cultivation broth of *B. velezensis* IP22 obtained in the validation experiment in the laboratory-scale bioreactor. Bacterial spot causal agents used for in planta testing were two *X. euvesicatoria* strains (PL1 and PL2) isolated from leaves of the diseased pepper plants. The resulting symptoms of pepper bacterial spot in the tested plants were recorded five weeks after a preventive treatment with *B. velezensis* IP22 cultivation broth and inoculation with pathogens. The results of in planta testing are summarized in Table 5 and presented in Figure 9.

In plants treated with *B. velezensis* IP22 cultivation broth, the symptoms of bacterial spot could be observed only at the spot where artificial inoculation with phytopathogenic *X. euvesicatoria* strains was performed (Figure 9), while the spreading of the disease symptoms outside of the inoculation spot on infected leaves was not noticed, which could be concluded from a significantly smaller value of leaf area covered by the necrosis caused by pathogen spreading (Table 5). In addition, further infection of other uninoculated leaves in the plants inoculated with phytopathogenic strains similarly was not observed (Figure 9). On the other hand, in the control plants that were not treated by *B. velezensis* IP22 cultivation broth, it could be observed that leaf necrosis had spread outside the inoculation area, covering 4–9 folds larger leaf area compared to the treated plants (Table 5). In addition, in untreated and inoculated control plants, symptoms of bacterial spot were also noticed on the leaves which were not directly inoculated, suggesting the disease spreading on 68–81% of total number of leaves, depending on the phytopathogenic isolate (Table 5). Furthermore, from the presented results, it could be also concluded that phytopathogenic isolate *X. euvesicatoria* PL 2 has caused more severe bacterial spot symptoms in pepper plants, which has also been confirmed by different levels of statistical significance when it comes to disease symptoms caused by PL1 and PL2 strains, but at the same time, its suppression by *B. velezensis* IP22 cultivation broth has been slightly more successful (Table 5, Figure 9).

## 4. Discussion

After molecular identification of the producing microorganism as *Bacillus velezensis* IP22 using 16S rDNA sequencing, the next step in development of the bioprocess aimed at production of the biocontrol agent effective against *X. euvesicatoria* phytopathogens was to formulate a medium suitable for cultivation of the producing microorganism and production of a highly-effective biocontrol agent. Since it was previously established that *B. velezensis* IP22 successfully utilizes glycerol as the carbon source for production of biocontrol agents and that combination of glycerol as the carbon source and yeast extract as the organic nitrogen source is optimal for maximization of its antimicrobial potential [46], the next step was modeling and optimization of the cultivation medium composition based on these nutrients’ content. Besides glycerol and yeast extract, concentrations of (NH_4_)_2_SO_4_ as the inorganic nitrogen source and K_2_HPO_4_ as the phosphorus source were also used as independent variables. The outputs, i.e., the monitored dependent variables were: inhibition zone diameters obtained by testing of antimicrobial activity of *B. velezensis* IP22 cultivation broth samples against *X. euvesicatoria* isolates PL1 and PL2, as well as residual content of glycerol, total nitrogen, and total phosphorus in the cultivation broth at the end of *B. velezensis* IP22 cultivation. Application of statistical modeling, using the second degree polynomial models, has showed satisfying results since the obtained models were statistically significant at the level of 99%. RSM (response surface methodology) has also revealed some useful insights into interactions of nutrients used in the cultivation medium and their effects on inhibition zone diameter, as the main dependent variable corresponding to *B. velezensis* IP22 antimicrobial activity against *X. euvesicatoria*. The RSM results have indicated high demand of the producing microorganism for nitrogen and phosphorus sources, which are mostly utilized for biomass growth and multiplication due to synthesis of cell components, such as phospholipids, proteins, and DNA [47], in order to achieve maximal antimicrobial activity against *X. euvesicatoria*, while the initial content of glycerol as the carbon source could be kept near minimal value of the examined range (10–60 g/L).

A further step in bioprocess development was optimization of the cultivation medium composition in terms of concentration of the main nutrients (carbon source, organic and inorganic nitrogen source, and phosphorus source), in order to produce a sufficiently effective biocontrol agent with maximized antimicrobial activity to be used against *X. euvesicatoria*. In this study, optimization was carried out using the desirability function method. The first optimization set had only one goal: to maximize the inhibition zone diameter of *B. velezensis* IP22 cultivation broth samples against *X. euvesicatoria*, i.e., to maximize antimicrobial activity of the producing microorganism. The optimized values of nutrients’ content, as well as the predicted value of inhibition zone diameter were in accordance with previously discussed results obtained using RSM: minimal concentration of glycerol and almost maximal concentration of yeast extract, (NH_4_)_2_SO_4_, and K_2_HPO_4_ from the examined range are required to obtain maximal inhibition zone diameter. However, the predicted values of residual nutrients’ content using the optimized cultivation medium composition were still very high, indicating possible presence of these nutrients in bioprocess effluents, which could represent a major problem for safe and sustainable disposal or treatment of bioprocess wastestreams [48]. Therefore, the second set of optimization of cultivation medium composition had the following goal defined: minimization of residual nutrients’ content, along with maximization of *B. velezensis* IP22 antimicrobial activity. The results of the second optimization set have shown a possibility to decrease initial nutrients’ content in the cultivation medium for 16.74%, 30.30%, and even 86.81% in the case of glycerol, yeast extract, and K_2_HPO_4_, respectively. On the other hand, the (NH_4_)_2_SO_4_ content should be increased for 11.52%, compared to the results of the first optimization set, probably due to decrease of nitrogen content arisen from a reduction of initial yeast extract concentration. These results indicate the possibility to significantly reduce the cost of the cultivation medium, contributing to the reduction of the total bioprocess cost. When it comes to the predicted values of residual nutrients’ content, it can be seen that they were reduced compared to the first optimization set: glycerol by 31.22%, total nitrogen content by 42.5%, and total phosphorus content by 95.32%. The most significant achievement in this particular case is the reduction of residual phosphorus content, considering that phosphorus, originating from wastestreams disposed in the natural aquatic ecosystems, is the main nutrient responsible for eutrophication [49]. Nevertheless, it can also be noticed that the predicted value of the inhibition zone diameter, and therefore, also the desirability function value, were reduced. The reduction of 9.47% in the inhibition zone diameter value, however, should not present a major loss of antimicrobial activity compared to the savings and benefits that could be achieved using the cultivation medium of the optimized composition, where further techno-economic analysis of the bioprocess should be employed to address the overall bioprocess cost-effectiveness.

In order to validate the obtained models for the selected dependent variables (inhibition zone diameter and residual glycerol, total nitrogen, and total phosphorus content) and the optimized values of nutrients’ content in the cultivation medium, as well as concurrence of the predicted values of dependent variables with their actual values, validation experiments were carried out by cultivating *B. velezensis* IP22 in a laboratory-scale bioreactor with working volume of 2 L. Monitoring of the *B. velezensis* IP22 cultivation course has revealed a typical bacterial growth curve, with the adaptation phase in the first 24 h, followed by the exponential phase (24th–60th hour) and the stationary phase observed after 60 h of cultivation. Consumption of nutrients by *B. velezensis* IP22 during the cultivation has followed biomass growth. However, it was interesting to notice that the trend of antimicrobial activity of cultivation broth samples against *X. euvesicatoria* has also followed the trend of biomass growth, where only slight changes in inhibition zone diameters could be observed during the stationary growth phase. These results indicate that cultivation duration could be reduced from 96 to 60 h, in order to reduce the bioprocess cost due to energy and labor savings, as well as the possibility to exploit bioprocess equipment more efficiently by increasing the number of production cycles per year. On the other hand, analysis of residual content of nutrients and inhibition zone diameters of cultivation broth samples against *X. euvesicatoria* at the end of the cultivation has revealed a significant match between observed values (1.36 g/L for glycerol content, 0.90 g/L for total nitrogen content, 0.15 g/L for total phosphorus content, and 60.55 mm for inhibition zone diameter) and the values predicted during the modeling of cultivation medium composition (Table 3). This matching, i.e., confirmation of validity of the obtained models for the inhibition zone diameter and residual content of glycerol, total nitrogen, and total phosphorus, indicates the applicability of the obtained models, as well as the optimized cultivation medium, for production of biocontrol agents effective against the causal agent of pepper bacterial spot *X. euvesicatoria* even at a larger production scale.

Since *B. velezensis* expresses several mechanisms of antimicrobial activity, such as competition for growth space and nutrients [18], production of volatile compounds [27], antimicrobial lipopeptides [17], and enzymes [18], one of the aims of this study was to investigate antimicrobial compounds produced by the *B. velezensis* IP22 strain isolated from fresh cheese, with proven in vitro antimicrobial activity against the causal agent of pepper bacterial spot *X. euvesicatoria*. With that purpose, samples of cultivation broth at the end of the cultivation in a laboratory-scale bioreactor were centrifuged, and supernatants were further analyzed using the HPLC-MS method. HPLC-MS analysis revealed presence of three lipopeptides putatively identified as members of the fengycin family and one lipopeptide putatively identified as a member of the locillomycin family, by comparing the obtained *m*/*z* values with literature data, as discussed in the following sentences. The fengycin family consists of lipopeptides with dominant antifungal activity, while the most probable mechanism of fungicidal action is disruption of cell membrane structure [50]. Fengycins with saturated fatty acid chains were detected in the *m*/*z* range of 1435.8–1505.8, while fengycins with unsaturated fatty acid chain with one double bond were detected in the *m*/*z* range of 1433.8–1489.8 [39]. Toral et al. [40] identified fengycin B with 16 C-atoms in the fatty acid chain at *m*/*z* 1491.8 [M+H]^+^. Fengycin with 16 C-atoms in the fatty acid chain has also been identified at *m*/*z* 1463.8 [M+H]^+^ [41]. Fengycins, which contain alanine at the sixth position in the peptide chain, have been identified at *m*/*z* values of 1471.9 [M+Na]^+^, 1485.9 [M+Na]^+^, 1499.9 [M+Na]^+^, for the compounds with 15, 16 and 17 C-atoms in the fatty acid chain, respectively [42]. According to de Fillippi et al. [43], fengycins A have been identified in *m*/*z* range of 1464–1506 [M+H]^+^, while fengycins B have been detected in the *m*/*z* range of 1462–1490 [M+H]^+^. Adeniji et al. [44] identified fengycins in the *m*/*z* range of 1449.9–1515.9. Recent study has confirmed antibacterial effect of fengycins against *Xanthomonas axonopodis* pv. *vesicatoria* by causing alterations on the cell surface and loss of intracellular content [51]. On the other hand, lipopeptide putatively identified as locillomycin was detected at *m*/*z* value of 1146.8, representing a relatively novel family of lipopeptides which exhibit antibacterial and antiviral activity, but also limited antifungal activity [45,52].

In order to assess suitability of the *B. velezensis* IP22 cultivation broth produced in the previously explained way to be used as a biocontrol agent against pepper bacterial spot, in planta tests with pepper plants were performed. Plants treated by the biocontrol agent and artificially inoculated using *X. euvesicatoria* strains PL1 and PL2, as well as positive and negative controls, were cultivated and monitored for symptoms of bacterial spot for five weeks. The preventive treatment of pepper plants with *B. velezensis* IP22 cultivation broth has significantly contributed to the suppression of phytopathogenic *X. euvesicatoria* strains, as well as to reduction of disease symptoms spreading. Differences in plant coloration between treated and untreated plants can be explained by promoting effects that *B. velezensis* has on plants as a part of its modes of action involved in antimicrobial activity, which is in agreement with recent studies on this agent [53,54,55,56] and which is yet to be investigated in our future studies. The results of in planta assaying suggest the high potential of biocontrol agents based on *B. velezensis* IP22 produced in the previously described way under the optimized production conditions at a laboratory scale in suppression of pepper bacterial spot caused by *X. euvesicatoria* strains. Hence, the validity of the proposed bioprocess solution for production of pepper bacterial spot biocontrol agent was confirmed, opening a new chapter of possibilities for scale-up of this bioprocess to a pilot and an industrial scale.

## 5. Conclusions

Results presented in this study indicate significant potential of *B. velezensis* IP22 to be used as a biocontrol agent for bacterial spot of pepper. Composition of cultivation medium for biotechnological production of biocontrol agents based on *B. velezensis* IP22 was optimized in order to maximize biocontrol activity against the tested *X. euvesicatoria* strains, but also to minimize residual content of nutrients in the cultivation broth in order to reduce costs of biotechnological production and make step further towards a sustainable production process. Validity of the optimized medium composition was confirmed in the experiments performed in the laboratory-scale bioreactor. The validation experiment has also given a useful insight into the possibility to reduce bioprocess duration from 96 to 60 h, which would contribute to reduction of the overall bioprocess cost. In planta experiments have confirmed the results of in vitro assays and significant potential of *B. velezensis* IP22 cultivation broth to be used in suppression of pepper bacterial spot. In addition, there is a wide spectrum of possible ways to improve the existing liquid formulation in order to increase its efficiency at the application site and to maintain viability of *B. velezensis* IP22 cells in a longer time period aimed at prolongation of the expiry date of the final product in which the guaranteed activity against the target phytopathogens should be delivered. Furthermore, the most pronounced mechanism of antimicrobial activity of the produced biocontrol agent is to be identified. Confirmation of the ability of *B. velezensis* IP22 to synthesize antimicrobial lipopeptides has opened a new chapter of possibilities to optimize cultivation medium composition in such order to achieve maximal concentration of the desired antimicrobial compounds in the resulting cultivation broth which will be lately applied as a biocontrol agent. Further research will be aimed at investigation of biocontrol activity of the produced agents based on *B. velezensis* IP22 in real application conditions, i.e., in the field, as well as at defining the optimal application method, dosage, and timing.

## Figures and Tables

**Table 1:** Experimental plan in the phase of in planta antimicrobial activity assaying.

Variant	*Xanthomonas* Isolate	Treatment	Plant Mark
Uninoculated untreated control–negative control	-	-	1
Inoculated untreated control–positive control	PL1	-	2A
Inoculated untreated control–positive control	PL2	-	2B
Inoculated treated plant	PL1	*Bacillus* IP22 cultivation broth	3A
Inoculated treated plant	PL2	*Bacillus* IP22 cultivation broth	3B

**Table 2:** ANOVA (analysis of variance) for the selected responses in the phase of modeling of medium composition for cultivation of *B. velezensis* IP22.

Response	SS	DF	MS	F	*p*-Value	R^2^ (%)
Inhibition zone diameter (mm)	39076.79 ^a^ 7.57 ^b^	15 ^a^ 12 ^b^	2605.12 ^a^ 0.63 ^b^	4127.72	<0.01	94.94
Residual glycerol content (g/L)	17270.84 ^a^ 218.02 ^b^	15 ^a^ 12 ^b^	1151.39 ^a^ 18.17 ^b^	63.37	<0.01	94.89
Residual total nitrogen content (g/L)	27.56 ^a^ 0.12 ^b^	15 ^a^ 12 ^b^	1.84 ^a^ 0.01 ^b^	191.00	<0.01	95.68
Residual total phosphorus content (g/L)	89.67 ^a^ 2.18 ^b^	15 ^a^ 12 ^b^	5.98 ^a^ 0.18 ^b^	32.91	<0.01	90.39

SS—sum of squares, DF—degree of freedom, MS—mean squares, R^2^—coefficient of determination. ^a^ model, ^b^ residual.

**Table 3:** Results of the optimization of medium composition for cultivation of *B. velezensis* IP22.

	First Set		Second Set	
Factor	Goal	Optimized Value	Goal	Optimized Value
Glycerol content (g/L)	in range	12.01	in range	10.00
Yeast extract content (g/L)	in range	4.06	in range	2.83
(NH_4_)_2_SO_4_ content (g/L)	in range	2.69	in range	3.00
K_2_HPO_4_ content (g/L)	in range	8.11	in range	1.07
Response	Goal	Predicted Value	Goal	Predicted Value
Inhibition zone diameter (mm)	maximize	66.75	Maximize	60.43
Residual glycerol content (g/L)	in range	2.05	Minimize	1.41
Residual total nitrogen content (g/L)	in range	1.60	Minimize	0.92
Residual total phosphorus content (g/L)	in range	2.78	Minimize	0.13
Desirability	1.00		0.77	

**Table 4:** Antimicrobial compounds putatively identified by the HPLC-MS (high pressure liquid chromatography–mass spectrometry) in the supernatant of *B. velezensis* IP22 cultivation broth.

Putatively Identified Compound	Retention Time (min)	Peak Area	*m*/*z*
Fengycin	8.883	62897	1461.8
Fengycin	1.603	12026	1489.7
Fengycin	8.96	60938	1490.9
Locillomycin	1.574	44514	1146.8

**Table 5:** The results of in planta antimicrobial activity assaying of the produced biocontrol agents based on *B. velezensis* IP22 against *X. euvesicatoria* strains, the causal agents of pepper bacterial spot.

Plant	Infected Leaves (%)	Leaf Necrosis (%)
1	0.00 ± 0.00 ^a^	0.00 ± 0.00 ^a^
2A	68.33 ± 1.67 ^b^	31.97 ± 0.14 ^b^
2B	80.95 ± 2.38 ^c^	44.43 ± 3.34 ^c^
3A	14.84 ± 0.55 ^d^	7.51 ± 1.76 ^d^
3B	14.84 ± 0.55 ^d^	5.09 ± 0.09 ^e^

A—*X. euvesicatoria* PL1, B—*X. euvesicatoria* PL2. 1—negative control, 2—positive control, 3—treated plants. Superscript letters (^a–e^) represent different levels of statistical significance. Values in the same column marked with the same superscript letter are at the same level of significance with confidence level of 95% (Duncan’s test).

## Supplementary Materials

Supplementary materials can be found at https://www.mdpi.com/2076-2607/8/10/1463/s1. Table S1. PCR conditions for identification of pathogens using the species-specific primers for *X. euvesicatoria.* Table S2. Box-Behnken experimental plan for optimization of medium composition for *Bacillus* IP22 cultivation.
