# Supplementary material for: Pepper Bacterial Spot Control by Bacillus velezensis: Bioprocess Solution
Source: Microorganisms. 2020 Sep 24;8(10):1463. doi: 10.3390/microorganisms8101463 (PMC7656301; doi:10.3390/microorganisms8101463)
Supplement: Supplementary file 1 [file microorganisms-08-01463-s001.pdf]

Supplementary Material

Pepper bacterial spot control by *Bacillus velezensis*: bioprocess solution

Ivana Pajčin [1,*], Vanja Vlajkov [1], Marcus Frohme [2], Sergii Grebinyk [2], Mila Grahovac [3], Marija Mojićević [4] and Jovana Grahovac [1,*]

[1] University of Novi Sad, Faculty of Technology Novi Sad, Bulevar cara Lazara 1, 21000 Novi Sad, Serbia; paj@tf.uns.ac.rs, vanja.vlajkov@uns.ac.rs, johana@uns.ac.rs
[2] Technical Institute of Applied Sciences Wildau, Hochschulring 1, 15745 Wildau, Germany; marcus.frohme@th-wildau.de, sergii.grebinyk@th-wildau.de
[3] University of Novi Sad, Faculty of Agriculture, Trg Dositeja Obradovića 8, 21000 Novi Sad, Serbia; mila@polj.uns.ac.rs
[4] University of Belgrade, Institute of Molecular Genetics and Genetic Engineering (IMGGE), Vojvode Stepe 444a, P. Fah 23, 11010 Belgrade, Serbia; marijamojicevic@imgge.bg.ac.rs
* Correspondence: paj@tf.uns.ac.rs; Tel.: +381-65-5344196, johana@uns.ac.rs; Tel: +381214853682

Content

Table S1. PCR conditions for identification of pathogens using the species-specific primers for *X. euvesicatoria*

Table S2. Box-Behnken experimental plan for optimization of medium composition for *Bacillus* IP22 cultivation

Table S1. PCR conditions for identification of pathogens using the species-specific primers for *X. euvesicatoria*

Phase	Temperature (°C)	Duration	Number of cycles
Initial denaturation	94	3 min	1
Denaturation	94	45 s	
Primer binding	64	50 s	35
Elongation	72	50 s	
Final elongation	72	10 min	1

Table S2. Box-Behnken experimental plan for optimization of medium composition for *Bacillus* IP22 cultivation

Experiment	Glycerol (g/L)	Yeast extract (g/L)	$(NH_4)_2SO_4$ (g/L)	K_2HPO_4 (g/L)
1	10	0	1.5	5.5
2	60	0	1.5	5.5
3	10	5	1.5	5.5
4	60	5	1.5	5.5
5	35	2.5	0	1
6	35	2.5	3	1
7	35	2.5	0	10
8	35	2.5	3	10
9	10	2.5	1.5	1
10	60	2.5	1.5	1
11	10	2.5	1.5	10
12	60	2.5	1.5	10
13	35	0	0	5.5
14	35	5	0	5.5
15	35	0	3	5.5
16	35	5	3	5.5
17	10	2.5	0	5.5
18	60	2.5	0	5.5
19	10	2.5	3	5.5
20	60	2.5	3	5.5
21	35	0	1.5	1
22	35	5	1.5	1
23	35	0	1.5	10
24	35	5	1.5	10
25	35	2.5	1.5	5.5
26	35	2.5	1.5	5.5
27	35	2.5	1.5	5.5
